# “There is more that unites us than divides us”. Optimizing talent transfer processes by clustering 34 sports by their task, individual and environmental similarities

**DOI:** 10.3389/fspor.2024.1445510

**Published:** 2024-11-01

**Authors:** Jan Willem Teunissen, Jelle De Bock, Dominique Schasfoort, Maarten Slembrouck, Steven Verstockt, Matthieu Lenoir, Johan Pion

**Affiliations:** ^1^ Institute for Studies in Sports and Exercise, HAN University of Applied Sciences, Nijmegen, Netherlands; ^2^ Department of Movement and Sports Sciences, Faculty of Medicine and Health Sciences, Ghent University, Ghent, Belgium; ^3^ UGent-imec ELIS Department, Research Group IDLab, Ghent University—IMEC, Ghent, Belgium

**Keywords:** machine learning (ML), talent transfer, sports, clustering, experiential knowledge, coaches, questionnaire

## Abstract

Sports are characterized by unique rules, environments, and tasks, but also share fundamental similarities with each other sport. Such between-sports parallels can be vital for optimizing talent transfer processes. This study aimed to explore similarities between sports to provide an objective basis for clustering sports into families by means of machine learning. An online survey was conducted, garnering responses from 1,247 coaches across 36 countries and 34 sports. The survey gauged the importance (0 = not important 10 = important) of 18 characteristics related to the sport and the athlete performing in that sport. These traits formed the basis for the categorization of a sport by means of machine learning, particularly unsupervised clustering, and the LIME feature explainer. Analysis grouped 34 sports into five clusters based on shared features. A similarity matrix illustrated the degree of overlap among sports. The application of unsupervised clustering emphasized the lack of a single overarching attribute across sports, marking a shift away from traditional clustering approaches that rely on a limited set of characteristics for talent transfer. The results highlight the importance of identifying common sports for talent transfer, which could prove advantageous in guiding athletes towards new sporting directions.

## Introduction

Many coaches claim that their sport is incomparable, highlighting its distinctiveness in terms of rules, environment (such as specific surfaces and equipment), tasks (like the use of hands), and the unique physiological and anthropometric demands placed on individuals to meet the sport's objectives. Additionally, each sport has its own required gear, culture, and unspoken norms that could also affect motor behaviour (1). Despite these differences, sports also share many similarities, such as skill sets as seen between futsal and football (2) or physiological demands like those seen in practice between ice skating and road cycling. Sports federations can leverage these similarities to streamline talent transfer processes (3), giving athletes a chance to find success in a new sport if they fall out of love with their current one or must stop due to injury (4). Yet, the genesis of many talent transfer or crossover initiatives between sports is the identification of certain shared characteristics among athletes, such as anthropometry, physical, and motor skills. For a talent transfer to be effective, it is crucial that, a significant number of similarities exist between the sports (5). This study aims to pinpoint the most substantial overlaps between sports, grouping them to aid coaches and federations in their exploration of potential transfer options between sports. An effective transfer can take place when the action scenarios between sports or activities share a considerable degree of similarity (6), enabling the athlete to progress more swiftly through the initial stages of learning (5) and potentially hastening the skill acquisition process in the new sport through skill transfer (7). This idea aligns with (8) Transfer of Learning concept, which advocates for the utility of shared skills and knowledge in new or different contexts. Athletes cross-over to a new sport and then capitalize on previous investments and are fast-tracked into the transfer sport (5, 7), using less time to compete at the highest level (9). A recent example is Petr Cech, who after his retirement from elite football as a goalkeeper, currently found a new challenge in playing (ice) hockey as a goalkeeper. Although this example represents a successful transfer, it did not result from a systematic approach to motivate athletes for a new or different sport. A systematic approach to talent transfer would certainly be valuable for federations to minimize talent loss and optimize medal opportunities (3).

The UK Sport's Girls4Gold program illustrates how sports federations formally employ the strategy of identifying similarities between sports to facilitate the talent transfer process more efficiently (3). This program specifically sought to enlarge the pool of talent for various sports by focusing on (female) athletes who were prepared for and suitable for transitioning to new sports. In this formal talent transfer program, the emphasis is typically on selecting potential candidates based on metrics such as skill, anthropometry, and physical capabilities, prior to the stages of talent confirmation and further development (4). Nonetheless, the decision to recommend a new sport based on these profiles tends to be somewhat intuitive. Questions arise as to which sport constitutes the best fit and whether a comprehensive set of factors is considered, rather than isolated attributes (e.g., speed, endurance, skill).

Teunissen et al. (10) proposed a novel method for pinpointing sports that are suitable for talent transfers. Their approach begins with the creation of comprehensive profiles for a range of sports to gain insights into the unique requirements for excelling in each sport. Following this, they recommend aligning these sport profiles with potential opportunities for athlete transfers. This approach aims for a deliberate alignment of athletes into new sports, based on shared characteristics, to facilitate a smoother transition. By concentrating on sports with significant commonalities right from the start, this strategy allows for targeted actions to support transfers (10), alongside identifying athletes whose capabilities match the criteria for successfully adapting to a new sport. Grouping sports by their similarities is anticipated to enhance the effectiveness of talent transfers, enabling coaches to identify the most suitable sports for transfers by capitalizing on their significant commonalities. However, simply classifying sports based on individual characteristics might provide a limited perspective, not fully encompassing the breadth of similarities that exist among sports.

Traditionally, clustering methods in sports have frequently depended on isolated characteristics, game concepts, opinions, or traditions, as seen in the grouping of racket sports. This classification is well-documented in the literature, where sports such as tennis, table tennis, squash, and badminton are categorized together as racket sports (11). This grouping is also reflected in practice, with many national tennis federations, for instance, incorporating padel into their activities (12). This categorization primarily hinges on the common element of racquet usage. This clustering concept concentrates on a single characteristic (e.g., skill type or equipment usage) to discern commonalities among sports. Nevertheless, the literature also explores alternative methods of grouping sports based on the types of skills they demand. Schmidt and Wrisberg (13) detail several methods for categorizing skills within sports, such as separating skills into discrete, serial, and continuous types; evaluating the mix of cognitive and motor requirements (for example, comparing chess to high jump); and distinguishing between open and closed skills in executing tasks specific to various sports. Consequently, Schmidt & Wrisberg (13) recommended grouping sports for talent transfer based on shared movement, conceptual, and perceptual elements. Adding to this framework, Baker (14) described a fourth dimension—physical conditioning—as another critical transferable element. Yet, the conceptual foundation of clustering sport for talent transfer is not robustly established, highlighting the need for a more comprehensive and systematic examination of the similarities across sports and the potential for grouping them based on the most significant overlap of the profiles of each sport.

Acknowledging the importance of established concepts and their influence on practical implementations, this research delves into the grouping of sports through a comprehensive examination of the interplay among environmental, task, and individual constraints, moving beyond the simplistic categorization based on single attributes. This study builds upon the foundational work of Teunissen et al. (10), venturing deeper into identifying similarities across sports by clustering them based on the most significant overlap. To achieve this, unsupervised statistical techniques from the field of machine learning are employed to decode the complex network of shared traits among various sports clusters. The goal is to categorize sports based on the extent of characteristic overlap, thereby enhancing the efficacy of talent transfer initiatives.

## Methods

The project has been conducted in accordance with recognised ethical standards and was approved by the local ethics committee of the Ghent University Hospital (N° 20171548; Ghent, Belgium). All data were analysed confidentially.

An online survey, based on the SportKompas I Need survey (15), was administered via SurveyAnyplace, with 1,247 coaches from 36 countries and representing 34 different sports completing the questionnaire. Pion's (15) SportKompas study includes a range of tests designed to assess anthropometric, physical, and motor performance, proving effective in talent detection and identification across various sports. The variables used in the SportKompas study served as the foundation for this survey.

All the participating coaches had official coaching diplomas (964 club level, 71 national level, 130 unable to classify) of their respective countries. The included sports were: Archery (*n* = 16), Athletics (running) (*n* = 17), Badminton (*n* = 97), Baseball/Softball (*n* = 12), Basketball (*n* = 38), Bowling (*n* = 17), Canoe/Kayak (*n* = 52), Canoe/Kayak (sprint) (*n* = 10), Climbing (*n* = 28), Cycling (*n* = 47), Diving (*n* = 18), Figure Skating (*n* = 25) Free running (*n* = 21), Futsal (*n* = 11), Gymnastics (*n* = 32), Gymnastics (trampoline) (*n* = 34), Handball (*n* = 22), Hockey (*n* = 14), Judo (*n* = 26), Karate (*n* = 26), Mountain biking (*n* = 15), Netball (*n* = 29), Pencak Silat (*n* = 28), Rowing (*n* = 16), Sailing (*n* = 11), Sepak Takraw (*n* = 52), Shooting (*n* = 34), Soccer (*n* = 127), Swimming (*n* = 63), Table Tennis (*n* = 85), Taekwondo (*n* = 15), Tennis (*n* = 96), Triathlon (*n* = 37), and Volleyball (*n* = 76).

The survey commenced with an introductory page capturing essential details including name, gender, age, and varying levels of coaching qualifications. Subsequently, coaches rated the significance of 18 specific task (*n* = 14), environmental (*n* = 3), and individual (*n* = 1) attributes pertinent to their respective sport on a scale from 0 (not important) to 10 (highly important). The 18 characteristics were: agility; balance; catching; core stability; endurance; flexibility; hitting; jumping; kicking; speed of movement; rhythm; climbing; overall strength; throwing; indoor/outdoor sport; contact/no-contact sport; individual/team sport and stature of the participants tall/small. Intraclass correlation coefficient revealed good test-retest reliability ranging from 0.827–0.860 and *p* < 0.001 (10).

**Figure 1:**
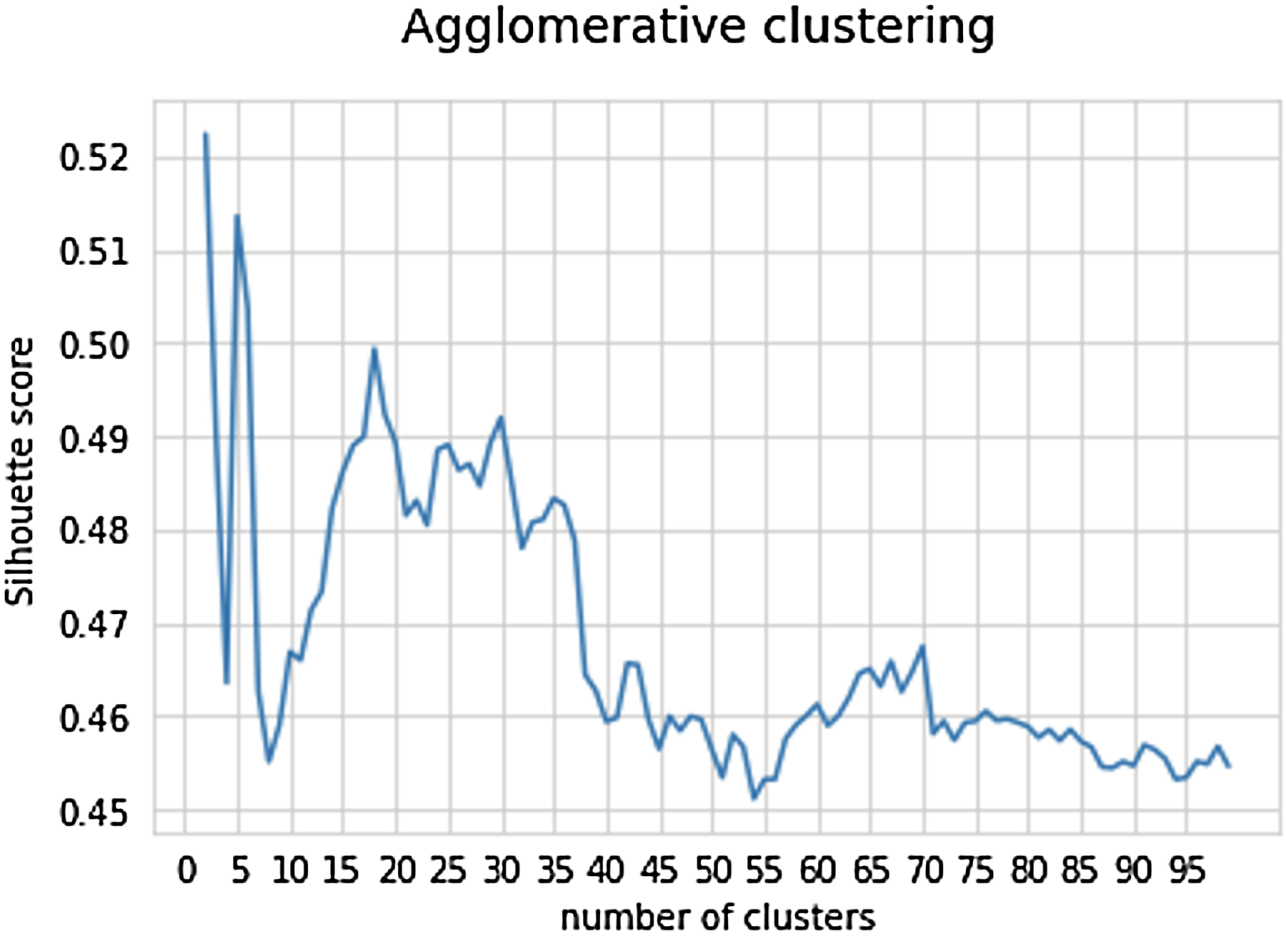
Shows the unsupervised analysis of the number of potential clusters for 34 sports based on the number of responses (*n* = 1,247) by agglomerative clustering. The number of clusters was limited from 2 to 100. The Silhouette score indicates how well a data point belongs to its assigned cluster (17).

**Figure 2:**
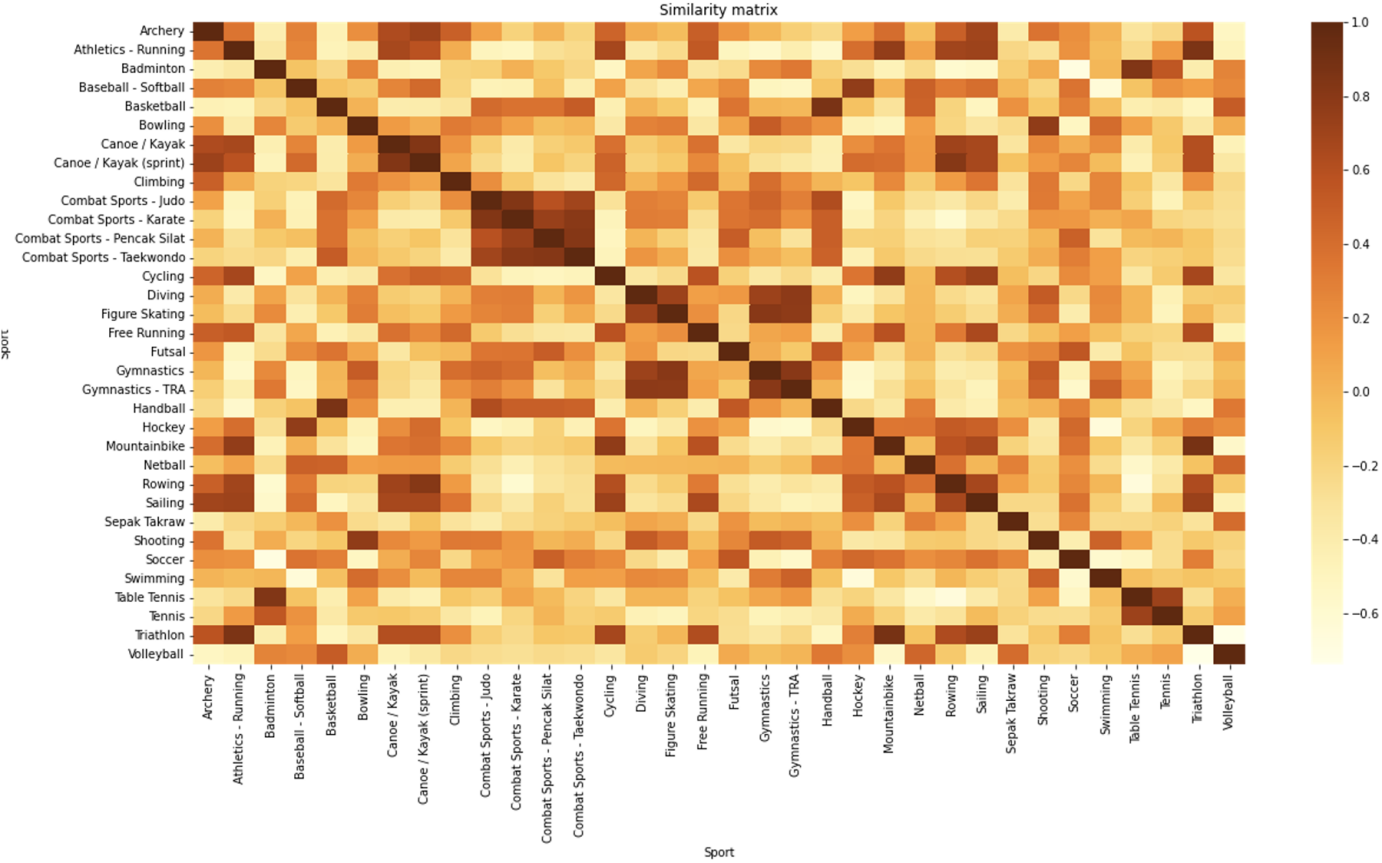
Cosine similarity matrix is a measure (unitless) that showcases the similarity among sports [−1,1], as determined by the LIME feature explainer's significance and effect for each respective sport. The color intensity indicates the degree of similarity between sports, the darker the shade, the greater overlap of the importance of the shared variables.

All statistical analyses were performed in Python programming language. At first, descriptive statistics were presented for 34 sports and the importance of its 18 characteristics and secondly, unique profiles per sport were obtained [see (10), for a detailed description of the first two steps of the methodology]. Thirdly, an unsupervised machine trained method was applied to search for similarities between sports. Agglomerative clustering of the processed data by Uniform Manifold Approximation and Projection (UMAP) technique was carried out to classify each sport in a group with similar importance of its characteristics. Agglomerative clustering is a common type of hierarchical clustering that uses the Euclidean distance between features. This algorithm was implemented using the Scikit-learn Python package (16). The input for the algorithm is the number of clusters that need to be distinguished. To find the optimal number of clusters a quality metric is needed to quantify how well data was separated in clusters. The silhouette score is such a metric that allows quantification of the clustering success. It uses the inter- and intra-cluster distances as a metric. The silhouette score quantifies the degree of similarity between an object and its own cluster (cohesion) vs. other clusters (separation). It is represented on a scale from −1 to +1, with a higher value indicating a strong match with its own cluster and a weak match with neighboring clusters (17). The silhouette score from the Python Scikit-learn package was analyzed with the number of clusters ranging from 2 to 100. Silhouette scores >.5 were addressed as potential number of clusters—as such, sports could be grouped in either 2, 5 or 17 clusters (see Figure 1). In line with current sport clustering models (14, 18) we have analyzed the data within 5 clusters. Fourthly, similarity clustering was performed using a CatBoost classifier [0.03 learning rate and Area-Under-Curve (AUC) evaluation metric], to construct a decision model for the classification of all 34 sports based upon the underlying importance of the characteristics. Finally, to gain a deeper insight in the model an additional library Local Interpretable Model-agnostic Explanations (LIME) was used to get feature importance for each of the classes’ predictions. In brief, the idea behind LIME is rather intuitive. It generates perturbations in the dataset presented to the model and looks at the impact of a perturbation of a feature in the dataset on the model's prediction outcome. To get overall feature importance per sport all samples of the dataset were ran through the LIME feature explainer. The explainer searches which of the features pushes the sample into the direction of its prediction. In contrast it also searches for features that have negative impact on the prediction (i.e., the lower the feature, the more chance that it gets predicted as that class). For a more detailed presentation of the relative similarities per sport a Cosine Similarity Matrix was generated based upon the Lime feature explainer (Figure 2).

## Results

Descriptive statistics and generated profiles of each of the 34 sports and their 18 characteristics are described in detail in Teunissen et al. (10).

**Figure 3:**
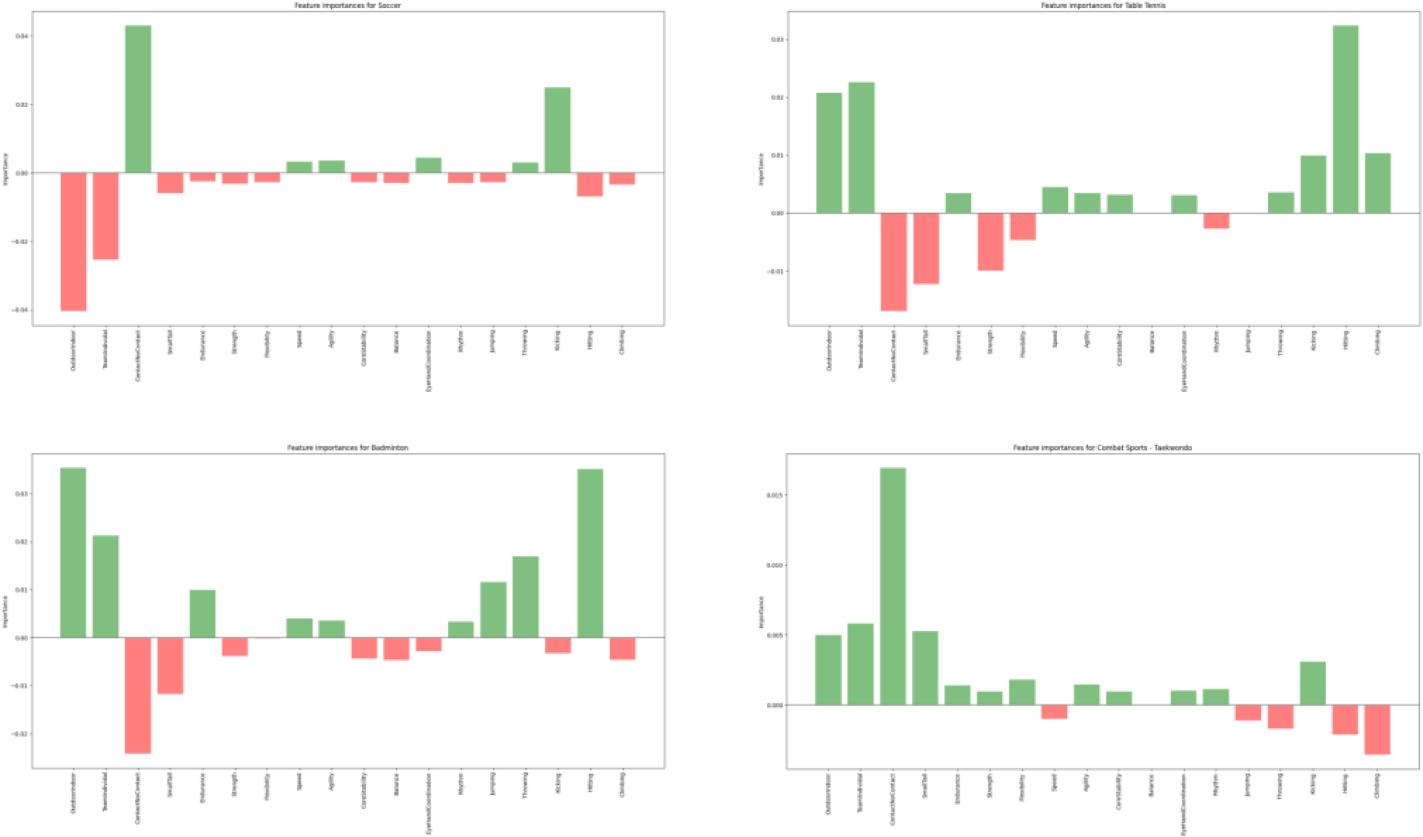
The different bars of the LIME feature explainer represent the importance of a variable for discriminating the individual responses from coaches to a specific cluster. Four examples are given for: Soccer, Taekwondo, Table Tennis, Badminton. The green and red colors indicate positive or negative contribution to the classes’ predictions.

A profile was drawn up for each sport by the LIME feature explainer. The metric is unitless and was computed for the comparison of feature influences for individual predictions. An example of four sports is given (see Figure 3—Lime Feature Explainer): Soccer, Table Tennis, Badminton and Taekwondo. The features per sport have either a positive or a negative effect on the prediction for each cluster and show the magnitude of the features for this prediction.

To indicate the degree of overlap between sports, a similarity matrix was created (see Figure 2—Cosine Similarity matrix). This heat map shows to what extent sports have similarities with each other based on the underlying feature importance's of each sport.

The unsupervised clustering of the LIME-generated profiles per coach response reveal that the 34 sports could be clustered in five groups based upon their features (see Figure 1). The initial clustering of the sports was performed by examining where each sport would best fit. Group 1 (*n* = 5): Futsal (100%), Baseball—Softball (92%), Soccer (92%), Sepak Takraw (60%), Pencak Silat (54%). Group 2 (*n* = 14): Diving (100%), Judo (96%), Figure Skating (96%), Free Running (95%), Athletics—Running (94%), Gymnastics—TRA (94%), Mountain bike (93%), Cycling (85%), Sailing (82%), Gymnastics (78%), Climbing (75%), Swimming (74%), Karate (65%), Triathlon (59%). Group 3: (*n* = 4): Table Tennis (94%), Tennis (78%), Badminton (64%), Taekwondo (60%). Group 4 (*n* = 5): Netball (97%), Handball (95%), Basketball (95%), Volleyball (68%), Hockey (43%). Group 5 (*n* = 6): Canoe/Kayak (sprint) (100%), Shooting (91%), Rowing (75%), Archery (75%), Canoe/Kayak (74%), Bowling (47%). However, not all sports had a 100% fit in their cluster as presented above. Some sports could be placed in two or more groups as shown in Table 1 in more detail.

**Table 1:** Classification of sports within the five different clusters.

Sport	Group 1	Group 2	Group 3	Group 4	Group 5
Futsal (*n* = 11)	100	0	0	0	0
Baseball—Softball (*n* = 12)	92	0	0	8	0
Soccer (*n* = 127)	92	2	6	0	0
Sepak Takraw (*n* = 52)	60	2	36	2	0
Pencak Silat (*n* = 28)	54	4	39	4	0
Hockey (*n* = 14)	36	21	0	43	0
Bowling (*n* = 17)	35	18	0	0	47
Badminton (*n* = 97)	34	2	64	0	0
Taekwondo (*n* = 15)	20	20	60	0	0
Volleyball (*n* = 76)	20	0	12	68	0
Tennis (*n* = 96)	18	4	78	0	0
Swimming (*n* = 63)	12	74	2	0	12
Rowing (*n* = 16)	12	12	0	0	75
Canoe/Kayak (*n* = 52)	10	16	0	0	74
Karate (*n* = 26)	8	65	23	4	0
Table Tennis (*n* = 85)	6	0	94	0	0
Gymnastics (*n* = 32)	6	78	9	6	0
Free Running (*n* = 21)	5	95	0	0	0
Handball (*n* = 22)	5	0	0	95	0
Judo (*n* = 26)	4	96	0	0	0
Climbing (*n* = 28)	4	75	7	0	14
Cycling (*n* = 47)	4	85	0	0	11
Basketball (*n* = 38)	3	3	0	95	0
Triathlon (*n* = 37)	3	59	0	0	38
Shooting (*n* = 34)	3	3	3	0	91
Netball (*n* = 29)	3	0	0	97	0
Diving (*n* = 18)	0	100	0	0	0
Figure Skating (*n* = 25)	0	96	4	0	0
Athletics—running (*n* = 17)	0	94	0	0	6
Gymnastics—TRA (*n* = 34)	0	94	3	0	3
Mountain bike (*n* = 15)	0	93	0	0	7
Sailing (*n* = 11)	0	82	0	0	18
Archery (*n* = 16)	0	19	6	0	75
Canoe/Kayak sprint (*n* = 10)	0	0	0	0	100

Table 1: Shows the unsupervised clustering results—to what extent the included sports fit [%] in the different groups based on their sport profile characteristics. Most of the included sports (31 out of 34 sports) can be clustered in multiple groups by their feature importance. The shaded percentages indicate the percentage ‘best fit’ of a particular sport.

## Discussion

The aim of this study was to search for similarities between environmental, task and individual characteristics of different sports by analyzing their unique profiles and cluster them to optimize talent transfer processes. The results show that the 34 sports could be grouped in five different clusters with an unsupervised machine learning method. Although all included sports could be grouped in one of five clusters, some of the included sports could also be classified in different clusters by the importance of its characteristics, therefore a more detailed analysis was carried out and revealed the relative similarities for all 34 sports in relation to one another.

In this study, sports have not been grouped based on certain similar characteristics, as is the case with e.g., the racket sports group. Rather, the grouping of sports emerged from an integrated evaluation of all relevant characteristics applied in this study and their significance for each sport by unsupervised methods in machine learning. Out of the 34 sports analyzed, 31 were classified into more than one group, illustrating the varied importance of their attributes within their profiles. The use of unsupervised clustering in this research highlights the absence of a single dominant attribute across the included sports, representing a departure from conventional clustering methods that depend on a narrow set of sport characteristics for talent transfer. Building on Ludwig Wittgenstein's theory of family resemblances, he contends that it's not possible to define concepts by isolating a single shared feature (essence), but rather, it is the collective set of features that defines a family—for instance, possessing blue eyes doesn't connect you with all individuals who have blue eyes as family (19). This perspective encourages a broader and more inclusive understanding of the composition of sports families, recognizing their varied and complex nature. It recognizes that sports may exhibit commonalities like environmental, task, individual similarities, yet no singular essence captures the entirety of sports. Instead, each sport is part of a broader, interrelated family, with connections among activities formed by a complex web of resemblances, moving beyond a strict structure of sameness.

The sports included in this study could be classified into five distinct groups, as the analysis reveals. The different clusters reveal potential opportunities for talent transfer based on the greatest possible overlap of the sports profiles. Especially for sports with a small talent pool, seeking talent transfer options to expand this pool is an opportunity (20, 21). These sports may see potential to pick up athletes who drop out of their sport and offer them new opportunities for possible success. For example, cluster two shows that gymnastics and springboard diving have a high degree of similarity, which is also discussed by Malina and Geithner (22). It would therefore be possible for springboard diving to look for athletes in gymnastics who want to make the switch, thereby enlarging their talent pool. This switch between gymnastics and springboard diving is supported by the fact that it has already occurred in practice as shown at the Athens Olympic Games in 2004. In the final of springboard diving 10 of the 12 female finalists had a gymnastics background (23). While the transfer from gymnastics to springboard diving looks promising both on paper and in practice, it is possible that this pathway might not be reciprocal (from springboard diving back to gymnastics). Certain sports, such as gymnastics, may serve as a “foundation” for other sports, facilitating transfers from gymnastics to related sports (22). However, the reverse transfer might not be as straightforward. Perhaps the number of skills required to perform in a sport play among others (e.g., specificity of skills) a role in defining more foundational sports (sports from which a transfer can occur) vs. sports where fewer skills seem necessary to be able to perform. Thus, the diversity of skills needed to engage in the sport might be an important factor for suggesting an efficient transfer.

In cluster four, handball and basketball are sports that show a high degree of similarity. However, it's speculative whether these sports exhibit reciprocity in their talent transfer process. In other words, both sports fit well within cluster 4 (both with 95% compatibility), yet whether the opportunities for talent transfer are equal in both directions remains a question. The discussion raised by Teunissen et al. (10) addresses this potential lack of reciprocity between the two sports. The absence of reciprocal profiles may stem from the differential emphasis on characteristics across sports. Characteristics that play a significant role in decision-making within the algorithm are given greater importance. A specific profile may inversely relate to characteristics in other profiles, aiming to optimize the classification process. Within the transfer of learning paradigm, this negative influence of skills can lead to adverse transfer between sports. Learning one skill often hinders the initial learning of another skill, resulting in negative transfer, as Edwards (24) noted. This effect of experience in one skill dampening performance in another skill could apply to basketball and handball, where the unique profiles may seem similar, but there could be different positive and potentially negative interrelations among the characteristics, possibly explaining the lack of reciprocity. These observations are consistent with the findings of Travassos et al. (2), suggesting that Futsal could act as a donor sport, enhancing the transfer of skills like ball control and technique to soccer. However, the crossover from soccer to Futsal might not be as evident. It is therefore, that the data should be interpreted with caution and further research should be conducted on the reciprocity of the talent transfer pathways between sports.

The impact of this method extends beyond competitive sports. Often, individuals first encounter the sport(s) that they will ultimately pursue within the setting of Physical Education (PE). Grouping sports can be extremely advantageous for physical education teachers. PE is vital as it provides the initial introduction to the sports in which many individuals will eventually excel. Given the impracticality for PE teachers to cover every sport or a large selection of sports and expect students to achieve proficiency in all, they must choose wisely to ensure the sports they select offer a broad spectrum of learning opportunities (25). Identifying similarities between sports and organizing them into clusters could allow PE teachers to narrow down the number of sports to a more manageable selection, aligning with the Teaching Games for Understanding (TGfU) approach (18). This strategy is designed to promote the transfer of skills across a group of sports sharing similar gameplay scenarios that demand analogous movements or tactical insights from the participants (26). However, the practicality of our proposed clustering method for PE teachers may be debatable, as it does not specifically emphasize the similarities between sports on a characteristic-by-characteristic basis for educational objectives. It cannot be affirmed that sports within the same cluster share a unique common characteristic, as previously mentioned in this discussion.

This study makes a significant contribution by examining a wide range of sports and collecting a substantial number of responses. The use of machine learning to identify overlaps between sports has proven effective in suggesting potential talent transfer opportunities. However, several limitations should be acknowledged. While a coach's expertise offers valuable insights into the demands of a sport (27), the actual process of transferring athletes between sports depends on the athlete's ability to adapt. Thus, further research is needed to determine whether the knowledge provided by coaches truly facilitates optimal talent transfer outcomes.

A critical next step in improving the talent transfer process is to validate the sport profiles developed through coach input against real athlete profiles. This entails examining whether the experiential knowledge of coaches accurately reflects the physical and technical capacities required for success in a specific sport. Previous findings support the idea that the unique demands of each sport shape an athlete's development (28) suggesting that more specialized training leads to increasingly distinct athlete profiles. However, tracking athlete performance characteristics should be conducted to verify these results in older age groups (i.e., elite athletes) as a benchmark for what is required to perform in a sport.

To strengthen the transfer process, field tests should be conducted to compare athlete performance with validated sport profiles. Successful alignment between athlete and sport profiles would enable a more efficient and targeted transfer process. Existing talent identification studies have already utilized general test batteries to highlight differences and similarities in athlete profiles across sports. For instance, Pion et al. (29) showed that effective talent orientation occurs when the profiles of combat sports align with the personal attributes of young athletes. Future research should build on this by comparing systematic approaches—such as aligning athlete and sport profiles—to current talent transfer methods like the UK Sport's Girls4Gold program, which uses a more intuitive process to match athletes to sports. This would allow for a more rigorous evaluation of whether data-driven models outperform intuitive talent matching in practice. A key example of the limitations of current approaches is Bullock et al. (9), where athletes were tested on a limited set of performance indicators (e.g., speed, power, team effort) for inclusion in the transfer sport of Skeleton. Although the selected parameters were intuitively linked to the sport, no prior needs-analysis was conducted to identify which specific factors were most critical for the sport of Skeleton and therefore talent transfer success. In conclusion, the effective design of talent transfer processes requires a comprehensive understanding of both the qualities an athlete possesses and the demands of the target sport. By employing validated tests to assess these demands, a more accurate ‘matching’ strategy can be developed to determine whether an athlete has the appropriate mix of attributes to facilitate a successful transfer. Moreover, beyond simply aligning profiles, factors such as athlete motivation play a crucial role in determining the success of the transfer (4). Ultimately, while comparing athlete and sport profiles offers a foundation for identifying potential transfer opportunities, the athlete's own willingness to make the transition remains a decisive factor.

Additionally, not every sport achieved a high response rate, influencing the volume of data collected. Despite the smaller datasets, we chose to proceed to establish meaningful categorizations. The computer-generated clusters depend on the assortment of sports currently included, meaning the classification could alter with the addition of new sports. This also applies to the model's training variables (characteristics included), currently based on SportKompas (15), which mainly concentrates on physical and motor skills. Yet, studies have underscored the significance of psychological traits in successful talent development and transfer (30, 31). Integrating different characteristics, like interpersonal or social skills, could result in varied cluster formations, suggesting that these clusters may not be as fixed as those found in existing frameworks.

In our study, we grouped coaches from various countries to create sufficiently large cohorts for meaningful analysis, recognizing the potential for differing perspectives across cultures. While we are aware that variations also exist within individual countries and organizations, some uniformity was necessary to effectively categorize the sports. This approach ensured coherent and statistically significant data but may have overlooked geographical, cultural and intra-organizational differences in coaching practices. Cultural differences significantly shape the training methodologies that coaches adopt, influenced by distinct historical, educational, and societal factors unique to each nation. For example, the United States and the United Kingdom emphasize balancing educational theories with practical applications, while Commonwealth nations integrate scientific, psychological, and social elements into a multidisciplinary coaching approach. In contrast, some regions focus more rigidly on performance metrics, often with less attention to educational or social considerations (32). These disparities affect both the content and delivery of coaching and the coach-athlete relationship, underscoring the importance of contextual awareness. Future research should explore how cultural and national origins influence coaching profiles, providing deeper insights for developing more tailored and effective training strategies.

In this study, we employed generic characteristics for each sport (e.g., pulling, jumping, endurance) based on the items from the SportKompas study (15). It is crucial to recognize, however, that sports also encompass specific features not addressed in this study, which contribute to the unique nature of each sport, such as particular techniques like the hook shot in basketball. This specific throwing technique distinguishes basketball from other throwing sports, but similar uniqueness can be found in the throwing techniques of other sports as well. When seeking sports that feature a hook shot for talent transfer purposes, this technique will be rarely encountered in the broader sports landscape. Due to its specialized nature and terminology, there is likely no direct transition from basketball to another sport. Nevertheless, by focusing on generic characteristics in talent transfer, it is possible to identify similarities between different sports. The unique aspects of each sport present challenges for comparison when specific characteristics are emphasized. Therefore, we opted to use generic characteristics to compare sports, understanding that this approach might result in the loss of sport-specific details when profiling the sports.

Moreover, clustering sports based on attributes, environment, or physiology may seem more intuitively valid than the proposed method in this study. Such conventional methods could lead to misconceptions about the nuanced relationships between sports, possibly preventing coaches from understanding foundational concepts or recognizing the practical implications for talent transfer programs, thereby overlooking opportunities to advise athletes on potential transfers.

## Conclusion

The categorization of sports into five distinct groups based on environmental, task, and individual characteristics provides a strategic framework for identifying potential talent transfer opportunities. This organization enables coaches to develop a nuanced understanding of the potential for transferring talent across different sports, which can aid in expanding their talent pools by identifying sports with significant profile overlaps. For example, the similarities between gymnastics and springboard diving indicate promising possibilities for talent identification and transfer. However, coaches should also consider the constraints of reciprocal talent transfers, as shown by the potential difficulties in moving talent from springboard diving back to gymnastics or between handball and basketball, as discussed. Acknowledging the varying significance of attributes within each sport's profile can help coaches facilitate more effective talent transfers, especially for sports with smaller talent pools that are looking to expand.

Future research should expand upon the foundations laid by this study to explore the holistic profiles of sports, incorporating a broader array of characteristics including psychological, interpersonal, and social skills. The inclusion of such traits could potentially alter the current clustering configurations and offer deeper insights into the nature of talent transfer. Investigating the specificity and diversity of skills required in various sports could further elucidate foundational sports and their potential as sources for talent transfer. Additionally, exploring the reciprocity of talent transfer pathways between sports with a comprehensive approach could uncover new avenues for maximizing athlete transfer across disciplines.

In closing, the title of this article features a quote from the IOC: “*There is more that unites us than divides us*” (33) which underscores the three core values of the Olympic movement—Excellence, Respect, and Friendship. Reflecting on this message from the aim of this study, sports share numerous commonalities and by looking beyond the confines of a single sport, it is possible to identify opportunities for athletes to transition successfully and efficiently to a different sport if the need arises. The purpose of this article was not to quantify the similarities and differences between sports. Instead, it aimed to illustrate that by identifying commonalities and thinking beyond the confines of a single sport, e.g., coaches and policymakers can better support athletes in making a potential transition to a new sport when necessary.

## Data Availability

The datasets presented in this article are not readily available because data can be made available by request. Requests to access the datasets should be directed to janwillem.teunissen@han.nl.
